# The new generation: quantum sensors

**DOI:** 10.1007/s00216-021-03554-7

**Published:** 2021-08-10

**Authors:** Günter Gauglitz

**Affiliations:** Institute for Theoretical and Physical Chemistry, Eberhard-Karls-University, Tübingen, Germany

The need for process control, pollution monitoring, and point-of-care diagnostics have pushed both the development of chemical and biochemical sensors and the improvement of classical analytical methods. The trend for sensors is towards miniaturization, parallelization in arrays, reduction of limit of detection, and the combination with chemometric methods in order to tackle new areas of analytical applications. After groundbreaking developments in optics and electroanalytics some years ago, the novelty of transduction methods in both biochemical and chemical sensors has worn off and the push for their innovation is flagging. Nevertheless, new strategies for recognition elements and an interest in being able to measure very low concentrations in even the smallest volumes in samples in order to monitor processes even in cells have increased the interest in advancing to new frontiers in sensing [1].

The recent success of quantum computing has influenced the development of an area in Industry 4.0—the development of quantum sensing [2]. The approach most commonly used for quantum computers is a quantum circuit based on a qubit which differs from the classical approach of a quantum state. In contrast to the classical approach, a qubit system is not in a defined state — it is averaged over the two states 0 and 1, and, according to quantum mechanics, can be a coherent superposition of both. The measurement of a qubit will destroy this coherence. Also, two particles which interact or share a special proximity can demonstrate a physical phenomenon such as quantum entanglement; even at a large distance, the quantum state of each particle cannot be described independently in this situation. Possible realizations of such systems are charged ions or spin qubits. Charged ions will be sensitive to electric fields, whereas spin-based systems will mainly respond to magnetic fields. Both, however, show a so-called intrinsic sensitivity, i.e., they exhibit a marked response to desired signals, but are only minimally affected by unwanted noise. With the spin approach, two adjacent carbon addends are removed from a diamond crystal, and one of them is replaced with a nitrogen atom. An excess electron of the nitrogen atom is placed into this defect center, and forms a so-called nitrogen-vacancy center (NV center). This electron has a spin, and the spin effect in the diamond can be optically initialized and read out. These spins follow the theory mentioned above with regard to certainty of state and/or quantum entanglement possibility [3]. A single NV center for quantum magnetometry or a single photon source can be produced. Such NV centers can be produced on a surface or even in a volume. NV centers allow the realization of sensors that surpass existing sensors in being extremely small, extremely low-noise, and ultra-sensitive sensors. Industry has recognized the possibilities of this new sensing principle for Industry 4.0 technology.

Therefore, apart from the application of quantum sensors in atomic clocks, superconducting quantum interference devices, and gravitational wave detectors, new ideas for applications in technology have been approached in recent years. The smallest fissures in the material or any distortions can be measured with the help of the magnetic field signature; for micro- and nano-electronic components, non-destructive examination techniques can be used. For example, it will be possible to map the subsurface infrastructure before commencing underground construction work. Besides these technical realizations, first applications in biosciences are considered, e.g., in the pharmaceutical industry, quantum sensors will allow determining the composition of capsule and tablet powders in an easier way. All this is possible even in situations where no good signals can be measured, and quantum effects can be used to detect details even in signal noise. This will enable the integration of such components in the smallest spaces on surfaces, in joints and gaskets, and in nanoreactors. For industry, the first prototypes are being merchandised. In analytics, only a few approaches have been realized yet.

Sensors based on NV centers, e.g., on nanodiamonds, will have a huge potential in biological applications, such as improvements in magnetic resonance imaging, the diagnosis of cardiovascular diseases by measuring the tiniest magnetic fields during metabolic processes of the cardiac tissues, or detection of extremely weak magnetic fields produced in metabolisms of living cells. Thus, NMR signals from multiple nuclear species in a sample volume of approx. 20 nm^3^ can be measured, which allows extending the use of NMR techniques to measurements inside cells [4]. This can even provide new information on cellular structures and processes. In first publications in journals of analytical chemistry, the tracking of the total redox status and oxidative stress in cells and tissues using electron-paramagnetic resonance with small-sized coated quantum dots (QDs) is also called a quantum sensor [5]. The coated QDs are transformed from the paramagnetic radical form into a diamagnetic hydroxylamine form and vice versa, depending on the nitroxide status. However, the definition of QDs as quantum sensors might require further discussion as to whether these “macro” particles follow the quantum technology with quantum entanglement. Another recent review handles perspectives in NV-assisted biosensing, discussing potential electric and magnetic field sensing. Evidence is given for a switch from conventional electrophysiological techniques to NV sensors, and for the possibility to measure neuronal signals [6]. Advantages in optically detected magnetic resonance techniques (ODMR) based on NV sensors will result in interesting applications for the examination of cardiac cells and tissues. Finally, in a sensor journal, the salinity sensitivity of the proposed sensor in seawater is described as being at least one order of magnitude lower than that of traditional optical fiber surface plasmon resonance sensors. Single photons as an input source are used for exciting surface plasmon polaritons [7]. Here also the applicability of the original definition of quantum effect is questionable.

A literature search shows that analytical journals have not yet published papers on real quantum sensing, whereas even in physics, such devices and applications have been described for several years. A lack of theoretical insight and of instrumentation might prevent application in analytical science. However, these new sensors based on quantum mechanics will revolutionize the application of sensors in many areas. Small and highly sensitive sensors, allowing high parallelization in combination with artificial intelligence, will open new areas of analytics. The combination of microcenters with near-field effects, nanostructure, and plasmonics can be a future goal in sensing. Submission of such papers will follow the guidelines of a recent editorial published in ABC [8]. The journal will observe closely the quantum sensor development, and will be very happy to publish related papers.

## References

[1] Gauglitz G (2020). Critical assessment of relevant methods in the field of biosensors with direct optical detection based on fibers and waveguides using plasmonic, resonance, and interference effects. Anal Bioanal Chem.

[2] Jenet, A.; Trefzger, Ch.; Lewis, A.; Taucer, F.; van den Berghe, L.; Tüchler, A.; Loeffler, M.; Nik, S. #Standards4Quantum Making Quantum Technology Ready for Industry Putting Science into Standards, JRC Conference and workshop report 2020, https://standards4quantum_report.pdf

[3] Degen CL, Reinhard F, Cappellaro (2017). Quantum sensing. Rev Mod Phy.

[4] Holzgrafe J, Gu QS, Beitner J, Kara DM, Knowles HS, Atatüre M, Nanoscale NMR (2020). Spectroscopy using nanodiamond quantum sensors. Phys Rev Appl.

[5] Lazarova D, Semkova S, Zlateva G, Tatsuya H, Aoki I, Bakalova R (2021). Quantum sensors to track total redox-status and oxidative stress in cells and tissues using electron-paramagnetic resonance, magnetic resonance imaging, and optical imaging. Anal Chem.

[6] Petrini G, Moreva E, Bernardi E, Traina P, Tomagra G, Carabelli V, Degiovanni IP, Genovese M (2020). Is a quantum biosensing revolution approaching? Perspectives in NV-assisted current and thermal biosensing in living cells. Adv Quantum Technol.

[7] Zhao Y, Peng Y, Hu XG, Xia F, Zhao Q (2020). Beating the shot-noise limit with optical fiber quantum sensors for salinity measurement. Sensor Actuat B-Chem.

[8] Baeumner AJ, Cui H, Gauglitz G, Moreno-Bondi MC, Szunerits S, Woolley AT. Advancements in sensor technology with innovative and significant research publications: how to write that perfect paper? Anal Bioanal Chem. 2021. 10.1007/s00216-021-03417-1. 10.1007/s00216-021-03417-1 34061245

